# Multimodal liquid biopsy for early monitoring and outcome prediction of chemotherapy in metastatic breast cancer

**DOI:** 10.1038/s41523-021-00319-4

**Published:** 2021-09-09

**Authors:** Amanda Bortolini Silveira, François-Clément Bidard, Marie-Laure Tanguy, Elodie Girard, Olivier Trédan, Coraline Dubot, William Jacot, Anthony Goncalves, Marc Debled, Christelle Levy, Jean-Marc Ferrero, Christelle Jouannaud, Maria Rios, Marie-Ange Mouret-Reynier, Florence Dalenc, Caroline Hego, Aurore Rampanou, Benoit Albaud, Sylvain Baulande, Frédérique Berger, Jérôme Lemonnier, Shufang Renault, Isabelle Desmoulins, Charlotte Proudhon, Jean-Yves Pierga

**Affiliations:** 1 Circulating Tumor Biomarkers laboratory, INSERM CIC BT-1428, Institut Curie, Paris, France; 2 Department of Medical Oncology, Institut Curie, Paris and Saint Cloud, Paris, France; 3 UVSQ, Université Paris-Saclay, Paris, France; 4 Department of Biostatistics, Institut Curie, Paris, France; 5 INSERM U900, Institut Curie, Mines ParisTech, PSL Research University, Paris, France; 6 Department of Medical Oncology, Centre Léon Bérard, Lyon, France; 7 Department of Medical Oncology, Institut du Cancer de Montpellier, Montpellier University, IRCM INSERM, Montpellier, France; 8 Department of Medical Oncology, Aix-Marseille Univ, INSERM U1068, CNRS UMR7258, Institut Paoli-Calmettes, CRCM, Marseille, France; 9 Department of Medical Oncology, Institut Bergonié, Bordeaux, France; 10 Department of Medical Oncology, Centre François Baclesse, Caen, France; 11 Department of Medical Oncology, Centre Antoine Lacassagne, Nice, France; 12 Department of Medical Oncology, Institut Jean Godinot, Reims, France; 13 Department of Medical Oncology, Institut de Cancérologie de Lorraine, Vandoeuvre-Lès-Nancy, France; 14 Department of Medical Oncology, Centre Jean Perrin, Clermont-Ferrand, France; 15 Department of Medical Oncology, Institut Claudius Regaud, IUCT-Oncopole, Toulouse, France; 16 ICGex Next-Generation Sequencing Platform, Institut Curie, Paris, France; 17 R&D UNICANCER, UCBG, Paris, France; 18 Department of Medical Oncology, Centre Georges-François Leclerc, Dijon, France; 19 INSERM U934 CNRS UMR3215, Paris, France; 20 Université de Paris, Paris, France

**Keywords:** Predictive markers, Breast cancer

## Abstract

Circulating tumor cells (CTCs) and circulating tumor DNA (ctDNA) are two cancer-derived blood biomarkers that inform on patient prognosis and treatment efficacy in breast cancer. We prospectively evaluated the clinical validity of quantifying both CTCs (CellSearch) and ctDNA (targeted next-generation sequencing). Their combined value as prognostic and early monitoring markers was assessed in 198 HER2-negative metastatic breast cancer patients. All patients were included in the prospective multicenter UCBG study COMET (NCT01745757) and treated by first-line chemotherapy with weekly paclitaxel and bevacizumab. Blood samples were obtained at baseline and before the second cycle of chemotherapy. At baseline, CTCs and ctDNA were respectively detected in 72 and 74% of patients and were moderately correlated (Kendall’s *τ* = 0.3). Only 26 (13%) patients had neither detectable ctDNA nor CTCs. Variants were most frequently observed in *TP53* and *PIK3CA* genes*. KMT2C*/*MLL3* variants detected in ctDNA were significantly associated with a lower CTC count, while the opposite trend was seen with *GATA3* alterations. Both CTC and ctDNA levels at baseline and after four weeks of treatment were correlated with survival. For progression-free and overall survival, the best multivariate prognostic model included tumor subtype (triple negative vs other), grade (grade 3 vs other), ctDNA variant allele frequency (VAF) at baseline (per 10% increase), and CTC count at four weeks (≥5CTC/7.5 mL). Overall, this study demonstrates that CTCs and ctDNA have nonoverlapping detection profiles and complementary prognostic values in metastatic breast cancer patients. A comprehensive liquid-biopsy approach may involve simultaneous detection of ctDNA and CTCs.

## Introduction

Liquid biopsy, a minimally invasive approach, has shown in the last decades prominent validity for diagnosis, prognosis, and for monitoring treatment efficacy in different cancer types. Throughout the years, several studies have explored the validity of different circulating analytes, including circulating tumor cells (CTCs), circulating tumor DNA (ctDNA), cell-free RNAs (mRNAs, long noncoding RNAs, and microRNAs), extracellular vesicles, and proteins markers. However, CTCs and ctDNA remain the most widely studied circulating biomarkers so far.

CTCs, released from the primary tumor or metastatic lesions, can be detected by immunocytological techniques^1^ and have been reported to play a key role in cancer metastasis through intravasation into the bloodstream and dissemination at distant sites^2^. In early-stage breast cancer (BC) patients, CTC enumeration has been used as an independent and powerful biomarker for survival and relapse prognostication^3,4^. This was also demonstrated in patients with metastatic breast cancer (mBC) where a decrease in CTC counts 3–5 weeks after the start of a new line of therapy correlates with treatment efficacy^5–7^.

ctDNA, a fraction of total cell-free DNA (cfDNA), is released into the circulation by tumor cells undergoing apoptosis/necrosis or by active secretion and its biological role is currently not known^8^. Using next-generation sequencing (NGS) or droplet-based digital PCR (ddPCR) technologies, multiple studies have demonstrated the utility of ctDNA detection as a prognostic biomarker for patients’ survival in early-stage and metastatic BC patients^9–11^. Unlike tumor tissue biopsies, ctDNA analysis is spatially unbiased, which allows systematic investigation of tumor molecular alterations and improves the detection of potential actionable mutations. ctDNA profiling has been successfully used in breast cancer patients to explore intratumor clonal heterogeneity and early disease evolution^12–14^ or as a diagnostic tool for treatment selection^15,16^. The use of CTCs and ctDNA biomarkers, therefore, provides real-time assessment of tumor dynamics and represents a major tool for selecting the best therapy and for monitoring treatment efficacy^17^.

Until now, the clinical significance of CTCs and ctDNA biomarkers has mostly been assessed independently and only a limited number of studies have quantified both biomarkers in parallel using the same blood draws from the same patients. In the present study, we have investigated the clinical value of concomitant analysis of CTCs and ctDNA. As part of a large prospective biomarker study (COMET) conducted in HER2-negative mBC patients treated by first-line chemotherapy, CTCs and ctDNA were analyzed at two time points: prior to treatment and after four weeks of first-line chemotherapy. The detection profiles and prognostic values of these two circulating biomarkers were then compared to understand their overlap and complementary contribution in mBC patients’ management.

## Results

### CTC enumeration and ctDNA detection at baseline and their associations with patients’ characteristics

**Fig. 1:**
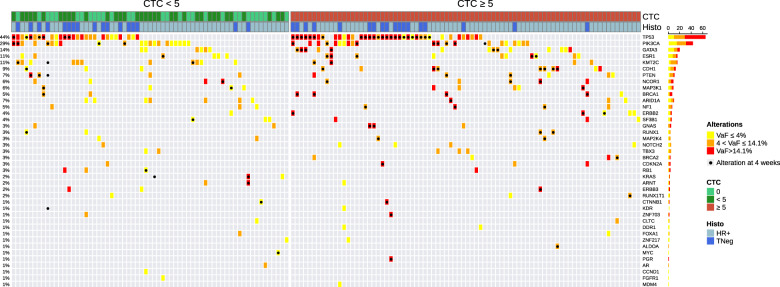
Variant-detection rate in cfDNA according to CTC numbers at baseline (*n* = 147). Variant-detection rates are displayed for each gene at baseline (*n* = 147 patients). VAF is colored by quartiles with higher VAF (>14%) in red, middle VAF (4 < VAF ≤ 14%) in orange, and lower VAF in yellow (≤4%).

Out of the 510 patients enrolled in the whole COMET study, analyses were performed on the 198 consecutive patients for whom CTC counts and ctDNA measurements were available at baseline (study flow chart in Supplementary Fig. 1). Clinical and pathological characteristics of this subset of 198 patients are displayed in Table 1. At baseline, 142 (72%) patients had one or more CTCs and 98 (49%) patients had five or more CTCs detected in 7.5 mL of blood (median: 4 CTC, range: 0–30,000). Variants were detected in 147 patients (Supplementary Table 1). A total of 251 single-nucleotide variants and 78 small indels were identified among the 54 genes targeted by the NGS approach (Supplementary Data 1). The median number of mutated genes per baseline sample was 1 (range 0–9) in the whole population and two in the 147 patients with detected ctDNA. Fifty-one (26%), 53 (27%), and 94 (47%) patients displayed zero, one, or more than one mutated gene in their baseline ctDNA sample, respectively. Variants were most frequently observed in *TP53* and *PIK3CA* genes (Fig. 1).

**Table 1:** Patient characteristics and association with circulating tumor biomarkers at baseline.

	All patients	(*n* = 198)					Patients with at least 1 variant detected in cfDNA (*n* = 147)					
		All patients	≥5CTC	*P* value	ctDNA detected	*P* value	TP53 variant detected	*P* value	PIK3CA variant detected	*P* value	ESR1 variant detected	*P* value
*N* patients		198	98		147		65		43		17	
Median age	(range)	57 (29–77)	56 (33–76)	0.29	56 (29–77)	0.45	52 (29–73)	0.03	55 (33–74)	0.60	60 (38–74)	0.07
Meno. status	Premenopausal	55 (28%)	32 (58%)	0.20	41 (75%)	0.99	24 (59%)	0.03	16 (39%)	0.04	3 (7%)	0.4
	Postmenopausal	141 (72%)	66 (47%)		104 (74%)		39 (38%)		27 (26%)		14 (14%)	
Histology	IC-NST and other	171 (88%)	79 (46%)	0.09	129 (75%)	0.50	60 (46%)	0.37	38 (30%)	0.56	15 (12%)	0.99
	Lobular	24 (12%)	16 (67%)		16 (67%)		5 (33%)		4 (25%)		1 (6%)	
Tumor grade	1 or 2	101 (56%)	46 (45%)	0.20	73 (72%)	0.66	22 (30%)	<10^−3^	21 (29%)	0.44	11 (15%)	0.2
	3	80 (44%)	45 (56%)		61 (76%)		38 (62%)		19 (31%)		4 (7%)	
Subtype	HR + HER2-	153 (77%)	77 (50%)	0.79	112 (73%)	0.67	35 (31%)	<10^−3^	38 (34%)	0.05	16 (14%)	0.07
	Triple Negative	45 (23%)	21 (47%)		35 (78%)		30 (86%)		5 (14%)		1 (3%)*	
PS	0	106 (54%)	50 (47%)	0.58	78 (74%)	0.95	33 (42%)	0.74	24 (31%)	0.83	8 (10%)	0.8
	1 or 2	92 (46%)	48 (52%)		69 (75%)		32 (46%)		19 (28%)		9 (13%)	
MFI	Synchronous	40 (21%)	20 (50%)	0.99	33 (83%)	0.25	20 (59%)	0.05	13 (39%)	0.13	1(3%)	0.12
	>6 months	154 (79%)	75 (49%)		111 (72%)		44 (40%)		29 (26%)		15 (14%)	
N of met. sites	1, 2	121 (61%)	55 (46%)	0.23	83 (69%)	0.04	28 (34%)	0.008	25 (30%)	0.92	12(15%)	0.3
	≥3	76 (39%)	42 (55%)		63 (83%)		36 (57%)		18 (29%)		5 (8%)	
Met. sites	Bone only	18 (9%)	9 (50%)	0.16	14 (78%)	0.05	4 (29%)	0.4	4 (29%)	0.79	1 (7%)	0.05
	Liver + /- other	99 (50%)	55 (56%)		80 (81%)		38 (48%)		20 (25%)		14 (18%)	
	Other	80 (41%)	33 (41%)		52 (65%)		22 (42%)		19 (37%)		2 (4%)	

CTCs and ctDNA detection rates are given for *n* = 198 patients at baseline. The detection rate of *TP53*, *PIK3CA* and *ESR1* genes is given for the 147 patients for which ctDNA was detected (i.e., with at least one variant detected). Several clinicopathological features were not available explaining the missing patients in some categories. cfDNA cell-free circulating DNA, ctDNA circulating tumor DNA, Meno. status menopausal status, IC-NST invasive carcinoma of no specific type, PS Performance Status, MFI metastasis-free interval, *N* of met. sites number of metastatic sites, *Variant reported as nonpathogenic.

Associations between the two blood tumor markers examined as dichotomized variables and patient characteristics are shown in Table 1. In the population of 147 patients with detectable ctDNA (i.e., with at least one variant detected), *TP53* variants were more often found in younger, premenopausal patients with triple-negative or grade-3 breast cancers, with a higher number of metastatic sites or synchronous metastasis. *PIK3CA* variants were more frequent but not restricted to HR-positive cancers and were mostly observed in premenopausal patients. Besides one variant with no known pathogenic function (P-123), *ESR1* variants associated with hormone-therapy resistance were restricted to the HR-positive cancers and more frequent in patients with liver metastases.

Moreover, considering blood biomarkers as continuous variables, we detected the associations between high fractions of ctDNA and high tumor grade, triple-negative status, altered performance status, and high number of metastatic sites. CTCs were higher in patients with lobular subtypes, altered performance status, and in the presence of liver or bone metastases (Supplementary Table 2).

### Correlation between CTC enumeration and ctDNA detection

**Fig. 2:**
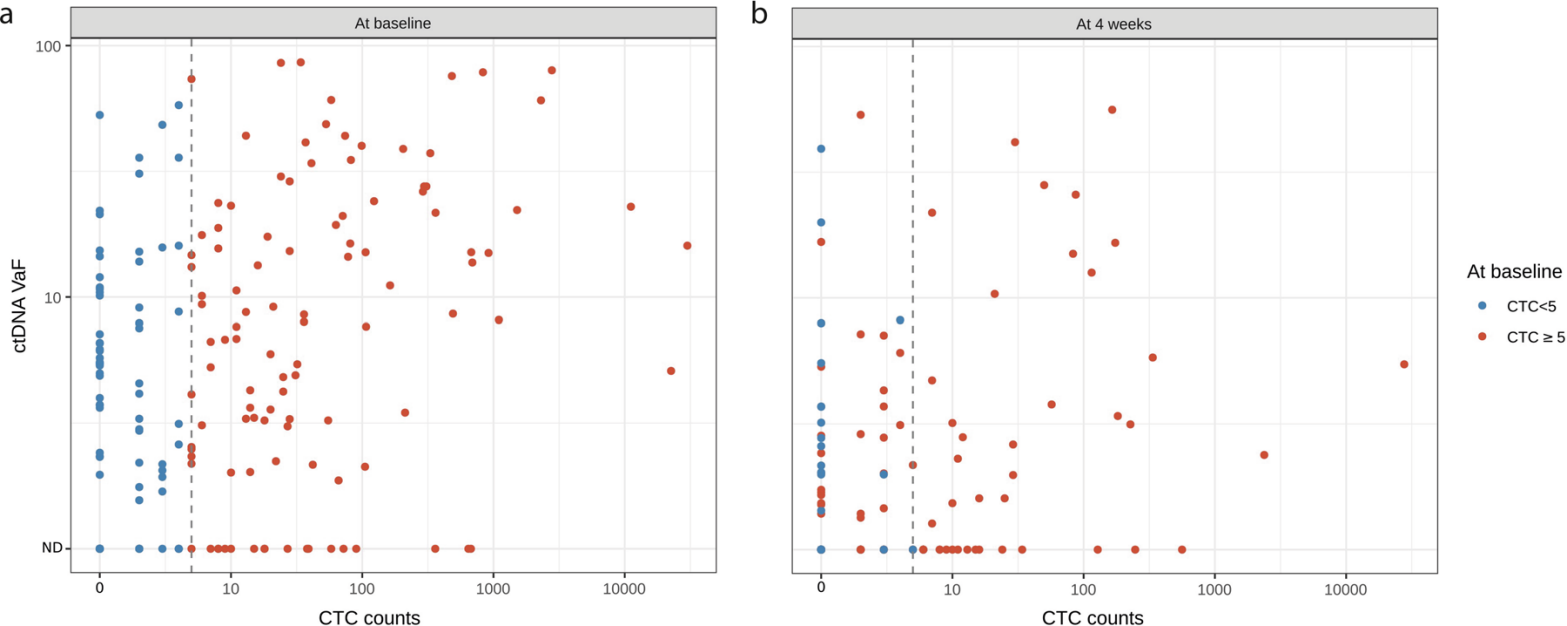
Correlation between CTC counts and ctDNA levels (logarithmic scale). Each dot represents ctDNA variant allelic fractions (VAF) as a function of CTCs counts (**a** at baseline, **b** at four weeks). ND ctDNA not detected. Dashed line highlights the 5 CTC thresholds.

In patients with detectable ctDNA (*n* = 147), a reporter variant was selected to analyze ctDNA-detection profiles and prognostic values in comparison with CTC measurements. The median allelic frequency of reporter variants at baseline was 7.6% (range 0.6–84.8%). CTC counts and ctDNA levels in each patient were moderately correlated (Fig. 2A; Kendall’s *τ* = 0.3, *p* < 0.001). At baseline, 82 (41%) patients had detectable ctDNA and five or more CTCs detected, 35 (18%) patients had no detectable ctDNA and less than 5 CTCs detected, and only 26 (13%) had neither ctDNA nor CTCs detected.

The number of CTCs detected in patients with certain mutated genes was then compared with the rest of the population with detectable ctDNA but no variant in the corresponding gene (Supplementary Fig. 2). We observed that the 16 patients with a *KMT2C*/*MLL3* variant had a lower CTC count (median 1.5 CTC, IQR 0–5) than the 131 patients with no *KMT2C*/*MLL3* variant detected (median 10 CTC, IQR 1–59.5, *p* = 0.007). Twenty patients had a *GATA3* variant and demonstrated an opposite trend, with an overall higher CTC count (median: 27 CTC, IQR: 4–74) than the 120 patients with no *GATA3* variant detected (median 6 CTCs, IQR 1–40; *p* = 0.07).

### Prognostic value of CTCs and ctDNA at baseline

**Fig. 3:**
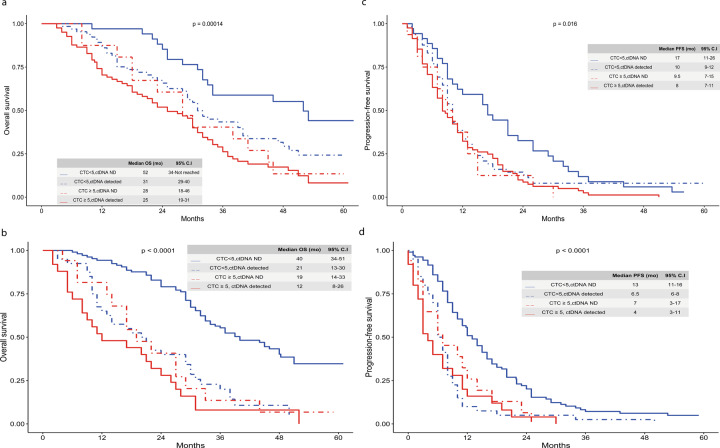
Overall survival and progression-free survival according to the combination of CTC counts and ctDNA levels. OS curves by blood biomarker status at baseline (**a**) and four weeks (**b**). PFS curves by blood biomarker status at baseline (**c**) and four weeks (**d**).

The prognostic impact of CTCs or ctDNA levels was first assessed at baseline independently. Both biomarkers were considered as dichotomized and continuous variables in univariate analysis. Shorter progression-free survival (PFS) and overall survival (OS) were observed in patients with higher CTC counts (Supplementary Fig. 3A–B; Supplementary Table 5) or higher ctDNA VAFs (Supplementary Fig. 3C–D; Supplementary Table 5). OS and PFS for combined CTC and ctDNA are shown in Figs. 3A and 3C (see also Table S5 for the corresponding CTC counts and ctDNA VAFs).

When considered as continuous variables, baseline ctDNA and CTC levels demonstrated a linear and log-linear impact, respectively, on the patient PFS and OS (Supplementary Fig. 4). There was no significant interaction between VAF impact on survival and individual gene (Supplementary Fig. 5).

Alterations in specific genes were also investigated to evaluate whether they have a prognostic impact on patients’ survival. This analysis was restricted to genes with variants detected in at least 15 patients (*TP53*, *PIK3CA*, *GATA3*, *ESR1*, *KMT2C*, and CDH1). We observed that patients with *TP53* variants displayed a worse overall survival (Supplementary Fig. 6). This was also significant in the subgroup of patients with HR-positive mBC. On the other hand, the detection of *ESR1* variants had no significant prognostic impact in this cohort, mostly composed of endocrine-resistant or HR-negative mBC (data not shown).

Finally, the added prognostic value of both CTCs and ctDNA to a prognostic model based on patient clinical and pathological characteristics was evaluated by different combinations. For PFS, the model included HR status, number of metastatic sites, tumor grade, and metastasis-free interval (CP model #1). For OS, the model included HR status, tumor grade, and metastatic site (visceral vs other, CP model #2). At baseline, the added value of both CTCs and ctDNA was higher when considered as continuous variables (linear or log-linear coding). At four weeks, biomarker levels gave a best fit when considered as dichotomized variables (<5 vs ≥5 for CTC and detectable vs not detectable for ctDNA). For PFS, we found that the increase in the prognostication accuracy of the model was not significant for CTC count at baseline (*p* = 0.2), while adding ctDNA VAF to the CP model #1 significantly increased the performance of the model (*p* = 0.004) (Table 2). Interestingly, for OS, both ctDNA and CTC levels at baseline significantly increased the prognostication accuracy of CP model #2 (*p* < 0.0001 and *p* = 0.008, respectively).

**Table 2:** Added value of blood tumor markers to prognostic models.

Comparison; timing & endpoint	Parameter tested for added value	LR test *p*-value
At baseline; progression-free survival		
CP model #1 vs CP model #1 + CTC_(bsl)_	CTC_(bsl)_	0.02
CP model #1 vs CP model #1 + ctDNA_(bsl)_	ctDNA_(bsl)_	0.0005
CP model #1 + ctDNA_(bsl)_ vs CP model #1 + ctDNA_(bsl)_ + CTC_(bsl)_	CTC_(bsl)_ if ctDNA_(bsl)_ known	0.2
CP model #1 + CTC_(bsl)_ vs CP model #1 + CTC_(bsl)_ + ctDNA_(bsl)_	ctDNA_(bsl)_ if CTC_(bsl)_ known	0.004
At baseline; overall survival		
CP model #2 vs CP model #2 + CTC_(bsl)_	CTC_(bsl)_	<0.0001
CP model #2 vs CP model #2 + ctDNA_(bsl)_	ctDNA_(bsl)_	<0.0001
CP model #2 + ctDNA_(bsl)_ vs CP model #2 + ctDNA_(bsl)_ + CTC_(bsl)_	CTC_(bsl)_ if ctDNA_(bsl)_ known	0.008
CP model #2 + CTC_(bsl)_ vs CP model #2 + CTC_(bsl)_ + ctDNA_(bsl)_	ctDNA_(bsl)_ if CTC_(bsl)_ known	<0.0001
At four weeks (landmark); progression-free survival		
CP model #1 + ctDNA_(bsl)_ vs CP model #1 + ctDNA_(bsl)_ + CTC_(4w)_	CTC_(4w)_	0.02
CP model #1 + ctDNA_(bsl)_ vs CP model #1 + ctDNA_(bsl)_ + ctDNA_(4w)_	ctDNA_(4w)_	0.01
CP model #1 + ctDNA_(bsl)_ + ctDNA_(4w)_ vs CP model #1 + ctDNA_(bsl)_ + ctDNA_(4w)_ + CTC_(4w)_	CTC_(4w)_ if ctDNA_(4w)_ known	0.1
CP model #1 + ctDNA_(bsl)_ + CTC_(4w)_ vs CP model #1 + ctDNA_(bsl)_ + CTC_(4w)_ + ctDNA_(4w)_	ctDNA_(4w)_ if CTC_(4w)_ known	0.07
At four weeks (landmark); overall survival		
CP model #2 + ctDNA_(bsl)_ + CTC_bsl_ vs CP model #2 + ctDNA_(bsl)_ + CTC_bsl_ + CTC_(4w)_	CTC_(4w)_	0.02
CP model #2 + ctDNA_(bsl)_ + CTC_bsl_ vs CP model #2 + ctDNA_(bsl)_ + CTC_bsl_ + ctDNA_(4w)_	ctDNA_(4w)_	0.04
CP model #2 + ctDNA_(bsl)_ + CTC_bsl_ + ctDNA_(4w)_ vs CP model #2 + ctDNA_(bsl)_ + CTC_bsl_ + ctDNA_(4w)_ + CTC_(4w)_	CTC_(4w)_ if ctDNA_(4w)_ known	0.02
CP model #2 + ctDNA_(bsl)_ + CTC_bsl_ + CTC_(4w)_ vs CP model #2 + ctDNA_(bsl)_ + CTC_bsl_ + CTC_(4w)_ + ctDNA_(4w)_	ctDNA_(4w)_ if CTC_(4w)_ known	0.05

CP model refers to the optimized prognostic models for PFS and OS. CP model #1 for PFS includes tumor subtype, grade, number of metastatic sites and metachronous relapses. CP model #2 for OS includes tumor subtype, grade and presence of visceral metastases. LR likelihood ratio, bsl level at baseline, 4w level at four weeks.

### CTC enumeration and ctDNA detection at four weeks

CTC enumeration and ctDNA levels in patients were obtained after four weeks of treatment (196 patients had CTCs quantified, 191 had ctDNA quantified, and 189 both, Supplementary Fig. 1). Out of the 196 patients with CTCs quantified at four weeks, 73 (37%) had one or more CTCs and 43 (22%) had five or more CTCs per 7.5 mL of blood (median: 0 CTC, range: 0–27,710). Regarding ctDNA, among the 191 patients assessed at four weeks, 124 (65%), 31 (16%), and 36 (19%) patients displayed zero, one, or more than one mutated gene, respectively. In patients with at least one variant detected at baseline, the median allelic frequency of reporter variants at four weeks was 0% (range 0–55%). Among the 189 patients with both CTC and ctDNA quantified at four weeks, 25 (13%) patients had detectable ctDNA and 5 or more CTCs and 94 (50%) had neither ctDNA nor CTCs detected. Overall, the detection rates at four weeks were significantly lower than at baseline for both tumor markers (both *p* < 0.0001) and were again moderately correlated (Fig. 2B; Kendall’s *τ* = 0.4, *p* < 0.001). Supplementary Table 3 depicts the different trajectories in terms of level changes for both biomarkers during the first four weeks of treatment. While CTC count and ctDNA levels had similar trajectories for 82 (43%) patients (both increasing or decreasing, highlighted in blue), an opposite trajectory (one marker becoming lower or undetectable while the other increased, highlighted in yellow) was observed in 28 (15%) patients.

### Prognostic value of CTCs and ctDNA after four weeks of treatment

In univariate analysis, CTCs and ctDNA levels at four weeks had a significant prognostic impact on both PFS and OS, when considered separately (Supplementary Fig. 7). OS and PFS for combined parameters are shown in Figs. 3B and 3D. We then evaluated the added prognostic value of CTCs or ctDNA level at four weeks to the optimal baseline prognostic model (based on CP model #1 and baseline ctDNA level for PFS and CP model #2, baseline ctDNA level, and baseline CTC count for OS, as displayed in Table 2). Both significantly increased the prognostication accuracy of the model in the same proportion. Finally, the best prognostic model for PFS included tumor subtype (triple negative vs other), grade (grade 3 vs other), ctDNA VAF at baseline (per 10% increase), and CTC count at four weeks (≥5CTC/7.5 mL) (Table 3).

**Table 3:** Multivariate analysis for PFS and OS.

Progression Free Survival		
	H.R; 95%C.I	*P*-value
Tumor subtype (triple negative vs other)	2.1; 1.4–3.1	0.0002
Tumor grade 3 vs other	1.5; 1.1–2.1	0.01
ctDNA at inclusion (per 10% increase)	1.2; 1.1–1.3	<0.0001
CTC count at four weeks (≥5CTC/7.5 mL)	1.7; 1.2–2.5	0.005

Overall survival		
	H.R; 95%C.I	*P*-value
Tumor subtype (triple negative vs other)	2.7; 1.7–4.3	<0.0001
Tumor grade 3 vs other	1.6; 1.1–2.3	0.01
ctDNA at inclusion (per 10% increase)	1.2; 1.1–1.3	0.0006
CTC count at four weeks (≥5CTC/7.5 mL)	2.3; 1.5–3.4	0.0001

Bootstrapping revealed moderate optimism of the model, with a decrease of the *c*-statistic from 0.68 to 0.67 and a calibration slope equal to 0.91. The variables retained in the best prognostic model for OS were tumor subtype, grade, ctDNA VAF at baseline (per 10% increase), and CTC count at four weeks (≥5CTC/7.5 mL). The bootstrapped c-statistic of the OS model was 0.73 (equal to the apparent c-statistic) and the calibration slope was 0.92. Overall, the time-dependent area under the curve (AUC) for 12-month PFS and OS status increased from 0.70 and 0.75 (models with baseline clinical variables only) to 0.76 and 0.85 (full PFS and OS models), respectively.

A normalization of the delta of ctDNA values and CTC counts between baseline and four weeks was also performed with the use of a Min–Max scaling (Supplementary Fig. 8). The HR associated with scaled ctDNA variation (dichotomized at the median) was 1.46–95%, C.I. 1.008–2.112, *p* = 0.045 for OS and 1.36–95% C.I. 0.97–1.89, *p* = 0.07 for PFS. The HR associated with scaled CTC count variation (dichotomized at the median) was 1.75–95%, C.I. 1.21–2.51, *p* = 0.003 for OS and 1.44–95%, C.I. 1.05–1.99, *p* = 0.03 for PFS.

## Discussion

As previously described^5,6^, CTCs were here detected in the peripheral blood of up to 70–80% of mBC patients. They were also associated with poor PFS and OS in multivariate analysis^18,19^. As the molecular analysis of CTCs is challenging, many studies concentrated on the enumeration of CTCs^20^. In contrast, ctDNA can be comprehensively analyzed with a variety of methods on archived plasma samples^11,21^. The proof-of-concept analysis showing that ctDNA is an informative, inherently specific, and highly sensitive biomarker for mBC was first described by Dawson et al. in 2013^22^. Among the analytes tested, ctDNA provided the earliest marker of treatment response compared with CA15-3 and CTC count in a series of 30 patients. The prognostic value of ctDNA detection was confirmed by a few larger studies^23,24^. Various techniques for ctDNA detection and quantification have since been reported^25–27^. COMET is the largest prospective study assessing the respective prognostic values of CTCs and ctDNA and their early changes during treatment in 189 mBC patients homogeneously treated with first-line chemotherapy. Prior retrospective studies on cohorts of mBC reported a prognostic impact for either one or both blood biomarkers, but the low patient numbers did not allow concluding on their correlation and clinical validity^22,28–30^. In a larger study, monitoring during treatment was limited^31^. More recently, this complementarity has been reported in the post-neoadjuvant setting at one time point after surgery^32^.

In this study, we observed a moderate correlation between CTCs and ctDNA detection at baseline and at four weeks. Interestingly, only 26 patients (13%) had neither ctDNA nor CTCs detected at baseline. This suggests that, similar to serum markers and CTCs^18^, combining ctDNA and CTCs could increase the number of patients assessable for marker detection and monitoring. The mutational landscape retrieved by ctDNA analysis was consistent with prior reports in HER2-negative BC, *PIK3CA* and *TP53* being the two most frequent variants. As expected, *TP53* variants were more often found in younger, premenopausal patients with triple-negative or grade-3 BCs, and with a higher number of metastatic sites. *PIK3CA* variants were more frequent but not restricted to HR-positive cancers. Activating *ESR1* mutations, which are selected as a mechanism of resistance to endocrine therapy^33^, were restricted to HR-positive cancers, and more frequently observed in patients with liver metastases. The large size of the COMET study allowed investigating associations between the tumor mutational landscape (retrieved by ctDNA analysis) and CTC-detection rates. We found that mBC patients with variants in the lysine (K)-specific methyltransferase 2 C gene (*KMT2C*, formerly known as mixed-lineage leukemia 3, *MLL3*, a histone methyltransferase involved in transcriptional coactivation) had a lower CTC count at baseline. KMT2C is frequently mutated in HR-positive breast cancer and associated with shorter PFS under anti-estrogen therapy^34^. In a gastric model, *KMT2C* inactivation promoted epithelial-to-mesenchymal transition and acquisition of stem cell-like phenotypes^35^. This may account for the significantly lower number of CTCs detected by the CellSearch system, which detects EpCAM and cytokeratin-positive epithelial CTCs. In addition, we observed a trend toward a higher CTC count in patients with *GATA3*-mutated BC (*p* = 0.07). Such association was previously reported in a smaller study^36^, and might be related to the putative role of GATA3 in BC cell migration and dissemination^37^. Finally, in our series, somatic variants in *CDH1* were seen in both infiltrating lobular and ductal carcinoma patients, although variants in this gene have been shown to be associated with lobular histology^38^. Higher CTC counts as a continuous variable were observed in patients with lobular cancers (*p* = 0.05). However, we did not confirm the correlation between *CDH1* variants and CTC count that was previously reported^36^.

We largely confirmed the validity of CTCs and ctDNA levels as prognostic markers before and during therapy. In addition to standard multivariate analyses, we measured whether one or both blood biomarkers significantly improve the accuracy of multivariate clinicopathological models. This well-established statistical method was already used to demonstrate the superiority of CTC count over serum markers^6^. Both approaches showed that more accurate prognostic information could be obtained when both blood biomarkers are considered. In detail, the best prognostic models for PFS and OS included ctDNA VAF at baseline (as a continuous variable) and CTC count at four weeks (as a dichotomized variable), highlighting that both blood biomarkers have nonoverlapping and complementary clinical validities. These results raise the question of using both markers for different purposes: (1) detection rates of both CTCs and ctDNA at baseline would help patient-survival prognostication and first-line strategy choice and (2) quantification at four weeks would help monitoring treatment efficacy. For this matter, CTC counts seem to outperform ctDNA. On the other side, ctDNA analysis at inclusion allows to better apprehend the tumor mutational landscape. While baseline CTC count demonstrated its utility in mBC patients as a decision tool before initiating a first-line treatment with chemotherapy or single-agent endocrine therapy^39^, CTC count monitoring during treatment has not yet demonstrated its clinical utility. In the SWOG 500 trial, patients whose mBC was resistant to first-line chemotherapy (as indicated by persistently high CTC count during therapy) did not benefit from the earlier introduction of a second-line chemotherapy^40^. Clinical utility of early ctDNA variation monitoring for mBC treatment remains also to be demonstrated^10^. Consensus recommendations by the American Society of Clinical Oncology have concluded that some ctDNA assays have clinical validity and utility as a noninvasive tool to assess the mutational landscape of a tumor^15^. However, none of these assays has been fully validated as an early monitoring tool during therapy.

One limitation of our study is that the CellSearch method, as explained above, may not detect CTCs that have lost epithelial features, but no other method has demonstrated its validity on a large scale at this time. Other limitations could be related to the small size of the NGS panel used to analyze ctDNA, potential inclusion of variants arising from clonal hematopoiesis, and limited sensitivity of the assay to detect variants with allelic frequency below 0.5%. However, higher sensitivity would not have changed the limited correlation observed between the CTCs and ctDNA as quantitative variables.

In that context, our study demonstrates that CTCs and ctDNA, which stem from very different biological processes, should be considered as yielding complementary and nonoverlapping clinical information.

## Methods

### Patients, treatment, and blood sampling

Patients used in this study were included in the multicentric prospective biomarker study “COMET” (NCT01745757) and were treated with weekly paclitaxel and bevacizumab as first-line chemotherapy. As per the 2012 European Medicine Agency label for bevacizumab, patients with triple-negative or hormone-receptor (HR)-positive, HER2-negative mBC were enrolled in this study. Other main inclusion criteria were written informed consent, age >18 years old, performance status (PS) of 0–2, life expectancy ≥3 months, and no prior chemotherapy for mBC. All patients received intravenous paclitaxel 90 mg/m^2^ on days 1, 8, and 15 with bevacizumab 10 mg/kg on days 1 and 15. Treatment was repeated every four weeks, until disease progression or unacceptable toxicity. The study was approved by an ethics committee (Comité de Protection des Personnes “Ile de France VII”) in June 2012 and registered under the following number NCT01745757. The primary endpoint of the study was the change of circulating endothelial cells (reported elsewhere^41^) and CTCs during therapy. Patients gave informed consent to use leftover plasma and tumor samples for further research, which allowed ctDNA analysis in this study although not mentioned in the initial research plan. Blood was drawn at baseline (prior to the start of chemotherapy) and at four weeks (prior to the start of the second cycle of treatment). Both markers were quantified in a central laboratory (The Circulating Tumor Biomarkers Laboratory, Institut Curie, France).

### CTC enumeration

CTC counts were determined using the standard CellSearch® assay. Blood collected into CellSave^®^ tubes was maintained at room temperature and processed within 96 h by experienced operators. Technical details of the CellSearch^®^ technique (Menarini Silicon Biosystems, Raritan, NJ) have been described elsewhere^1^.

### ctDNA analysis

For plasma isolation and cfDNA extraction, blood collected in EDTA tubes was processed on-site within 2 h of blood draw. Tubes were first centrifuged at 820 g for 10 min at RT. The supernatant was then centrifuged at 16000 g for 10 min at 4 °C and stored at −80 °C. cfDNA was extracted from 1 to 2 mL of plasma using the QIAamp circulating nucleic acid kit (Qiagen), following the manufacturer’s instructions. cfDNA was stored at −20 °C and quantified using a LINE1 real-time PCR assay as previously described (LINE1 forward primer: 5′-TCACTCAAAGCCGCTCAACTAC-3′; LINE1 reverse primer: 5′-TCTGCCTTCATTTCGTTATGTACC-3′)^42^.

For NGS library construction and sequencing, ctDNA detection was performed by capture-based targeted resequencing using a custom panel of 54 genes (Supplementary Table 1)^43–45^. Libraries were prepared using the Kapa HyperPrep kit (Roche) following the manufacturer’s instructions using xGen Dual Index UMI adapters from IDT, which contain one unique molecular identifier (UMI). Hybridization steps were performed on batches of 12 samples with the SeqCap EZ Accessory Kit V2 (Roche) and a custom panel of SeqCap EZ probes (Roche) covering 220 kb of the human genome. Sequencing was performed on an Illumina HiSeq 2500 instrument in batches of 84 samples using the Rapid Run mode PE 100. Matched archived tumor samples (available for 38 patients) were sequenced on an Illumina MiSeq instrument using a v2 cartridge PE 150.

For sequencing data analysis, raw reads were demultiplexed following IDT UMI recommendations and mapped to the hg19 human genome using bwa v0.7.15 (mem algorithm, default parameters^46^). UMI consensus sequences were generated and filtered using fgbio v0.5.0a-0 (com.fulcrumgenomics) with the following parameters: minimum mapping quality of 20 to keep an alignment, one error allowed per UMI. All UMIs supported by at least one read were selected and mapped to the reference genome. Only alignments intersecting the targeted sequence were kept using BedTools (v2.21.0^47^). Sequencing metrics for all libraries are described in Supplementary Data 2. Average coverage and consensus depths for cfDNA libraries were 2588X (range 121–9919) and 437X (range 6–1697), respectively. Average coverage for FFPE samples was 286X (range 58–616). Varscan2 (v2.4.1^48^) was then used to call both SNVs and indels in the alignment files considering or without considering UMI consensus sequences. For FFPE samples, UMIs were not considered. Only variants with an allelic frequency higher than 0.1% at a locus covered by at least eight reads were reported (mapping quality at 20 and base quality at 13). For FFPE samples, a minimum threshold of 5% VAF was used to filter out the variants. Variants were then annotated using Annovar (July 2017 version^49^) and the following annotations: refGene (October 2015 version), dbsnp147 and 1000 G (08/2015 version, all), Exome Sequencing Project (ESP6500, all), ExAc project, Cosmic (v70), ICGC (v21), and prediction algorithms such as SIFT, PolyPhen2, LRT, MutationTaster, PhyloP, and CADD (version ljb26). No technical filtering (based on metrics linked to the variants) was applied to cfDNA samples under the assumption that the confidence in variants called through the consensus of UMIs is high. The following filters were applied: (i) keep only exonic or splicing variant, (ii) discard variants frequent in the population (in at least one database with a VAF higher than 1%), and (iii) discard synonymous variants. A final curated list of nonsynonymous variants was obtained by manual filtering after visual inspection using IGV^50^. To increase the sensitivity of our approach (see below), whenever a variant was identified in FFPE tumor tissue, the same variant was systematically searched at baseline and week-four plasma samples with and without considering the UMIs. The same approach was applied to week four samples for variants identified in baseline plasma samples considering the UMIs. For patients with multiple variants detected in their cfDNA, a reporter variant was selected for monitoring. Prioritization was given in this order to variants (1) with positivity at baseline, (2) with positivity at four weeks, (3) with a COSMIC ID, (4) which were SNVs, and (5) presenting the highest VAF at baseline. All evaluations were carried out by qualified personnel with no knowledge of the patient’s clinical status.

Three quality controls of the ctDNA-detection assay were used to assess the sensitivity and the specificity of the custom-targeted resequencing approach.

(1) First, Seraseq™ ctDNA Mutation Mix v2 reference material, including 40 mutations in 28 genes, was tested, among which 12 (7 SNVs, three deletions, and two insertions) were covered by the panel (*PIK3CA*, *PTEN*, *KRAS*, *TP53*, and *GNAS*). Thirty-six samples comprising three replicates of Seraseq™ ctDNA Mutation Mix v2 at 0% (WT), 0.5, 1, and 2% VAF and at 3 different input quantities (8, 16, and 24 ng) were sequenced on an Illumina HiSeq 2500 instrument as described above. Average coverage was 3655X (3247–4226X) and consensus depth for UMIs was 817X (range 630–1036X). Average sensitivity was 97% at 2% VAF, 55% at 1% VAF, and 9% at 0.5% VAF for the UMI approach. When UMIs were not considered, average sensitivity was increased to 100% at 2% VAF, 94% at 1% VAF, and 77% at 0.5% VAF. Specificity was then assessed by quantifying detection rates for the same 12 variants in the WT reference material. We observed 100% and 97% specificity for the approach considering and not considering UMIs, respectively.

(2) Second, the detection rates of variants reported in Supplementary Data 1 were assessed in cfDNA obtained from 13 healthy individuals purchased from EFS (Etablissement francais du sang, France). Only 10 of the 329 variants found in patients were also detected in healthy controls. However, the higher VAFs observed in patient samples (average = 8.16%, median = 4.29% in patients vs average = 0.77%, median = 0.5% in healthy controls), together with other supporting data such as detection at the second time point or in tumor tissue, provided enough confidence for considering those variants as true-positive calls in patients samples.

(3) Finally, archived tumor material of patients, who gave written informed consent and treated at Institut Curie (available for 38 patients), was retrospectively sequenced to evaluate if variants identified in plasma samples were also observed in the tumor. These formalin-fixed paraffin-embedded (FFPE) tissues were macrodissected to increase tumor cell content prior to DNA extraction. DNA was extracted with the DNA FFPE Tissue Kit (Qiagen) and sequenced with the same targeted NGS method. Out of the 25 samples for which we obtained good-quality data, 24 (96%) displayed all or at least half of the variants observed in plasma DNA (Supplementary Data 1; P-94 and P-52 had 1/2 variant detected, P-129 and P-142 had 3/4 variants detected). The only patient (P-52) with no match between plasma and tissue had a single *ESR1* (p.D538G) variant detected in ctDNA, which was most likely absent from the primary tumor as these alterations are frequently acquired in patients under hormone therapy. The one variant not detected in P-142 was also in *ESR1* (p.D538G).

### Statistics

Associations between patient characteristics and blood biomarker levels when considered as dichotomized variables were studied using chi-square or Fischer’s exact test for categorical variables and Student’s *t*-test for age. When considered as continuous variables, Mann–Whitney, Kruskall–Wallis, or Student’s *t*-test were used for continuous data and Pearson correlation test for age. Associations between patient characteristics and *TP53*, *PIK3CA*, or *ESR1* gene variant status were studied using chi-square or Fisher’s exact test for categorical variables and Student’s *t*-test for age.

The correlation between CTC and ctDNA levels was assessed with the Kendall’s tau rank correlation. The comparison of the distribution of CTC levels according to the mutated gene status was performed with the Mann–Whitney test. We studied the genes for which variants were present in more than 10 patients (eight genes). No adjustments for multiple comparisons were made. Differences between baseline and week-four biomarker levels were studied with the use of the Wilcoxon signed-rank test.

PFS was defined as the time to disease progression or death of any cause. Overall survival was defined as the time to death, whatever the cause. Both PFS and OS were measured from the inclusion or the date of second sampling (four weeks) for the analyses based on biomarker levels at four weeks (landmark analyses). PFS and OS curves based on separate or combined CTC and ctDNA values were estimated by the Kaplan–Meier method.

The added value of biomarkers was assessed by comparing log likelihoods of models with and without biomarkers. We first fitted a model with baseline clinical and pathological characteristics (CP model) using a multivariable Cox regression analysis. We followed a backward stepwise-selection procedure. The stopping rule for exclusion of predictors was based on the Akaike information criterion (AIC). The variables tested in the model were age (<50 vs ≥50 years), performance status (0 vs ≥1), tumor subtype, tumor grade (≤2 vs 3), number of metastatic sites (≤2 vs 3), metachronous relapses, and presence of visceral metastases. The CP model was then compared with models in which biomarker levels were added (Table 2) by means of the Log-Likelihood ratio test. For this comparison, the added value of biomarkers was studied in two ways: after dichotomization (<5 vs ≥5 for CTC and detectable vs not detectable for ctDNA) and using restricted cubic splines (RCS) with four knots. For CTC, a log transformation was performed to deal with the skewed distribution. When RCS was used, we tested for nonlinearity in the biomarker effect. If no improvement in fit was found, the linear coding was used. To determine which form of the biomarker (continuous or dichotomized) should be kept in the model, models were compared with AIC. Optimal prognostic models were evaluated by conducting a bootstrap validation of model performance. Discrimination was assessed with the c statistic and calibration with the calibration slope. All tests were two-sided. A *P* value of less than 0.05 was considered to indicate statistical significance. All statistical analyses were done with R software, version 3.4.2 (R Foundation for Statistical Computing).

### Reporting summary

Further information on research design is available in the Nature Research Reporting Summary linked to this article.

## Supplementary information

Supplementary Information

Reporting Summary

Supplementary Data 1

Supplementary Data 2

Supplementary Data 3

Supplementary Data 4

Supplementary Data 5

## Data Availability

The ctDNA sequencing data are available in the NCBI Sequence Read Archive (SRA) under accession number PRJNA745047. PFS, OS data, CTC counts, and ctDNA values for individual participant after de-identification are publicly available in Supplementary Data 3 and Rdata. The sequence metrics used for ctDNA analysis are available in Supplementary Data 2.

## Code availability

The source R codes for the figures are available for nonprofit research use as Supplementary software.
