# Supplementary material for: Multimodal liquid biopsy for early monitoring and outcome prediction of chemotherapy in metastatic breast cancer
Source: NPJ Breast Cancer. 2021 Sep 9;7:115. doi: 10.1038/s41523-021-00319-4 (PMC8429692; doi:10.1038/s41523-021-00319-4)
Supplement: Supplementary file 1 — Supplementary Information [file 41523_2021_319_MOESM1_ESM.pdf]

Supplementary Materials

Supplementary tables

Supplementary Table 1: List of genes targeted for ctDNA detection

Supplementary Data 1: Sequencing metrics (Excel file)

Supplementary Table 2: Association between clinical factors, and CTC and ctDNA as continuous variables

Supplementary Table 3: CTC counts and ctDNA VAF (%) at baseline and 4 weeks for PFS and OS curves

Supplementary Data 2: List of variants detected in patients' plasma DNA (Excel file)

Supplementary Data 3: Clinical data and Blood biomarkers at baseline and 4 weeks (Excel file)

Supplementary figures

Supplementary Figure 1: Study flow chart

Supplementary Figure 2: CTC counts and ctDNA mutational landscape at inclusion

Supplementary Figure 3: Prognostic impact of CTC or ctDNA detection at baseline

Supplementary Figure 4: Hazard ratio according to blood biomarker levels

Supplementary Figure 5: Progression-free survival by VAF at baseline

Supplementary Figure 6: Overall survival according to *TP53* mutation status

Supplementary Figure 7: Prognostic impact of CTC or ctDNA detection at 4 weeks

Supplementary Figure 8: Variations of ctDNA values and CTC counts between baseline and 4 weeks

Supplementary software

The source R codes and Rdata for the figures

Table S1: List of genes targeted for ctDNA detection

Gene	Target
AR	All exons
ARID1A	All exons
ARNT	All exons
BRCA1	All exons
BRCA2	All exons
CCND1	All exons
CCNE1	All exons
CDH1	All exons
CDKN2A	All exons
CLTC	All exons
CYP19A1	All exons
DDR1	All exons
ERBB2	All exons
ERBB3	All exons
ERBB4	All exons
ESR1	All exons
FGFR1	All exons
FLT1	All exons
GATA3	All exons
GNAS	All exons
KDR	All exons
KMT2C	All exons
KRAS	All exons
MAP2K4	All exons
MAP3K1	All exons
MDM2	All exons
MDM4	All exons
MYC	All exons
NCOR1	All exons
NF1	All exons
NOTCH2	All exons
PBX1	All exons
PGR	All exons
PIK3CA	All exons
PPP2R2A	All exons
PTEN	All exons
RAD54B	All exons
RB1	All exons
RUNX1	All exons
RUNX1T1	All exons
SF3B1	All exons
TBX3	All exons
TP53	All exons
TP53BP1	All exons
ZNF217	All exons
ZNF703	All exons
ALDOA	Promoter region
CTNNB1	Promoter region
FOXA1	Promoter region
LEPROTL1	Promoter region
NEAT1	Promoter region
RMRP	Promoter region
TBC1D12	Promoter region
ZNF143	Promoter region

Table S2: Association between clinical factors and CTC and ctDNA as continuous variables

			CTC	P value	ctDNA	P value
		N	Median		Median	
Meno. status	Premenopausal	55	6	0.2	7.8	0.3
	Postmenopausal	141	4		3.1	
Histology	IC-NST and other	171	4	**0.05**	4	0.6
	Lobular	24	16		2.2	
Tumor grade	1 or 2	101	4	0.5	2.3	**0.01**
	3	80	7		7.9	
Subtype	HR+ HER2-	153	5	0.3	2.7	**0.004**
	Triple Negative	45	3		9.1	
PS	0	106	4	**0.003**	2.9	**0.01**
	1 or 2	92	7		6.8	
MFI	Synchronous	40	5	0.5	6.8	0.8
	>6 months	154	4		2.7	
N of met. sites	1-2	121	4	0.2	2.3	**<0.0001**
	≥3	76	7		8.7	
Met. sites	Bone only	18	5	**0.03**	2.6	0.07
	Liver +/- other	99	9		4.9	
	Other	80	4		2.6	

Meno. status: menopausal status; IC-NST: invasive carcinoma of no specific type; PS: Performance Status; MFI: metastasis-free interval; N of met. sites: number of metastatic sites.

Table S3: Blood biomarkers changes between baseline and 4 weeks.

**N patients**<br>**Marker levels**<br>**Measured at baseline / at 4 weeks**		**ctDNA**			**Total**
		bsl: ND<br>4w: ND	bsl: D<br>4w: ND	bsl: ND or D<br>4w: D	
**CTC**	bsl: <5 CTC/7.5mL<br>4w: <5 CTC/7.5mL	N=32	N=44	N=19	N= 95
	bsl: ≥5 CTC/7.5mL<br>4w: <5 CTC/7.5mL	N=6	N=25	N=21	N= 52
	bsl: < or ≥5 CTC/7.5mL<br>4w: ≥5 CTC/7.5mL	N=9	N=8	N=25	N= 42
**Total**		N=47	N=77	N=65	**N= 189**

bsl: baseline; 4w: 4 weeks. ND: not detected; D: detected. Background colors refer to changes of the two blood biomarkers in the same direction (blue, both increasing or decreasing) versus opposite direction (yellow, one increasing and the other decreasing).

Figure S1: Study flow chart

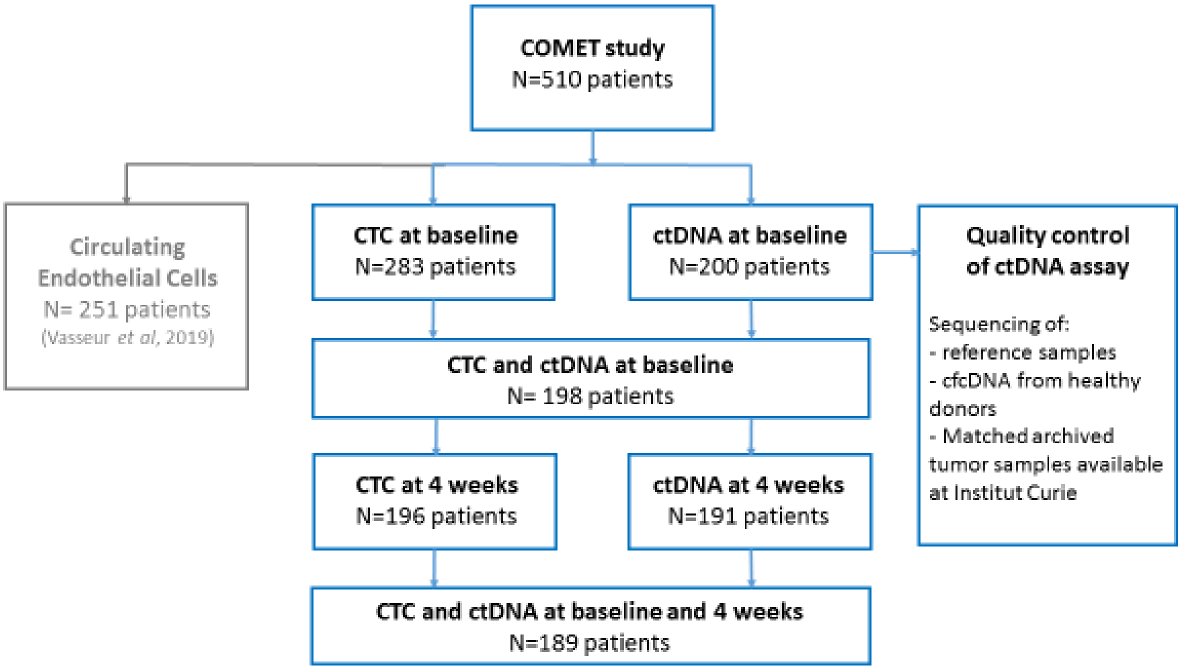

Figure S2: CTC counts and ctDNA mutational landscape at inclusion

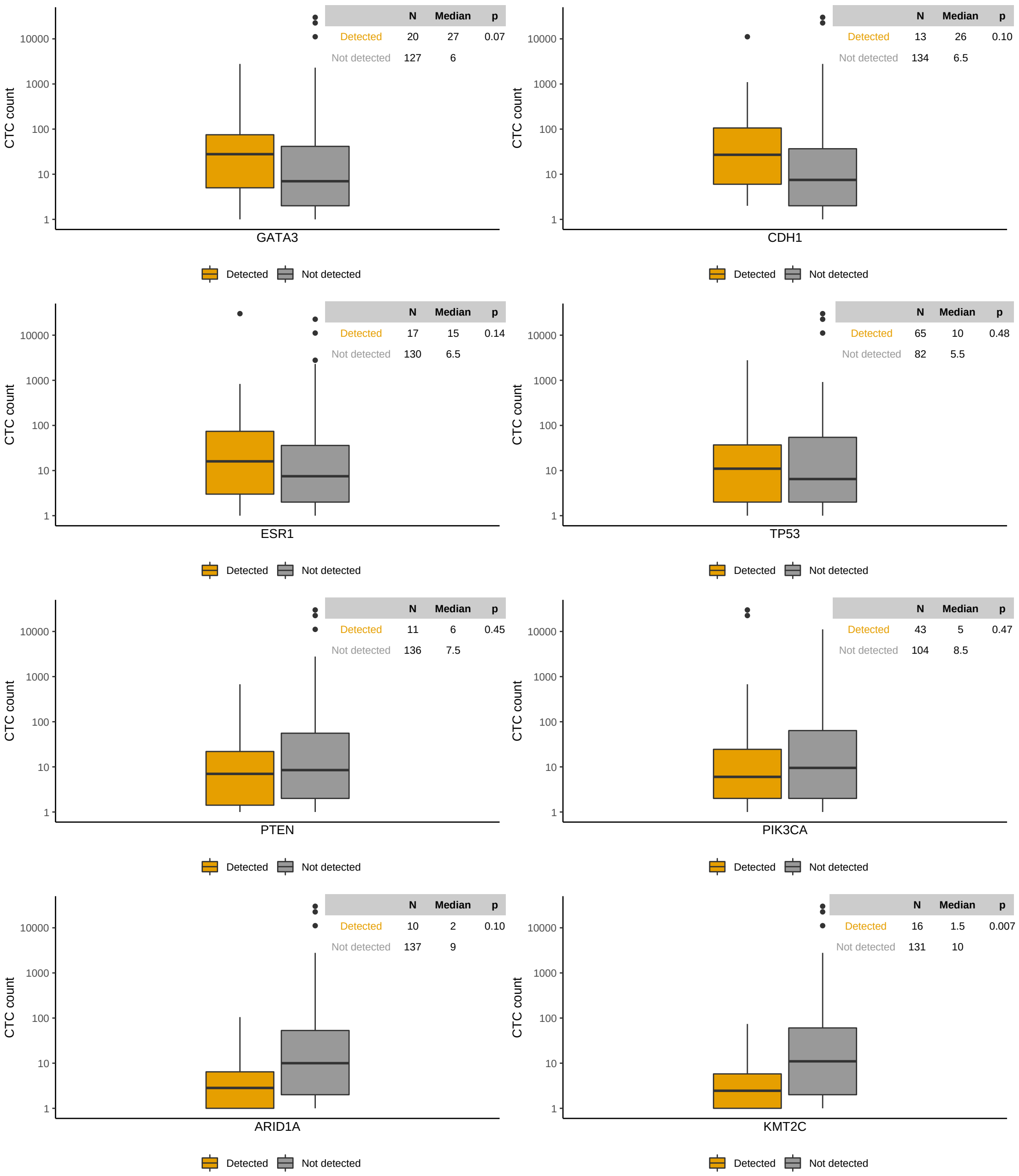

CTC counts distribution in patients with variants detected in *GATA3*, *CDH1*, *ESR1*, *TP53*, *PTEN*, *PIK3CA*, *ARID1A* and *KMT2C* compared to patients with detectable ctDNA but no variant in the corresponding gene. Graphs are shown in decreasing order for CTC counts median values in altered patients. This analysis was performed on genes mutated in at least 10 patients at baseline.

Figure S3: Prognostic impact of CTC or ctDNA detection at baseline

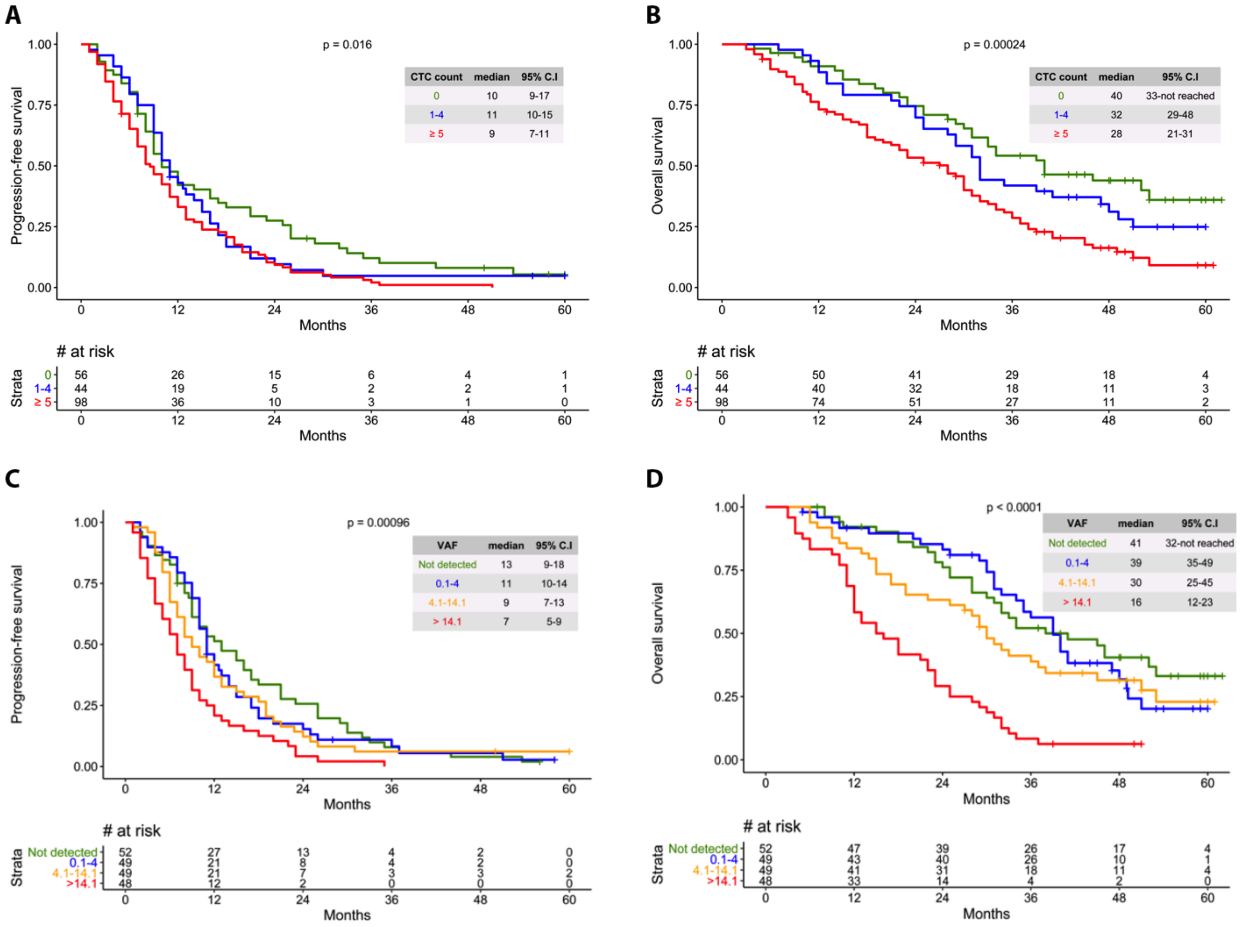

PFS (A) or OS (B) by CTC counts at baseline. PFS (C) or OS (D) by ctDNA levels at baseline.

Figure S4: Hazard ratio according to blood biomarker levels

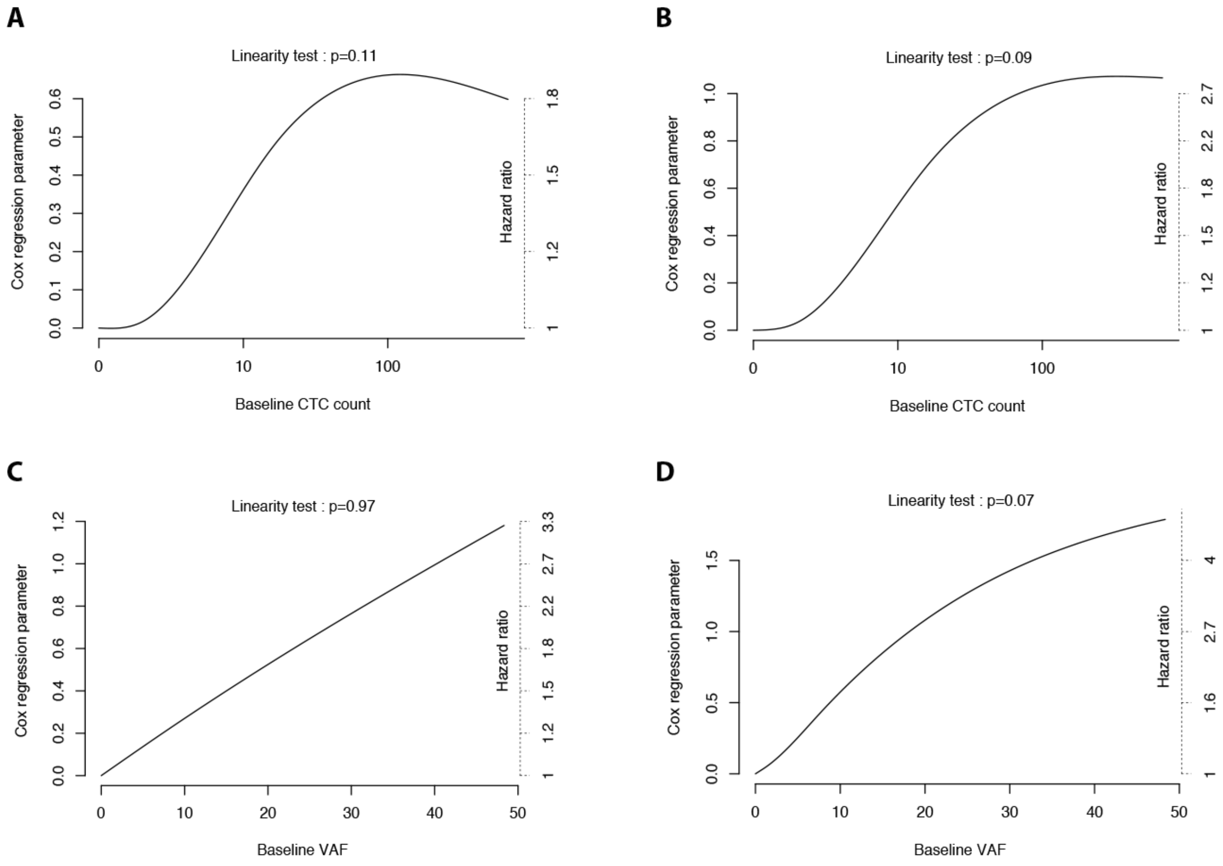

Plots of the estimated restricted cubic spline function relating CTC count and ctDNA levels to the Cox regression parameter.

(A) Cox regression parameter as a function of baseline CTC count (logged X-axis) for PFS. B: Cox regression parameter as a function of baseline CTC count (logged X-axis) for OS. C: Cox regression parameter as a function of baseline ctDNA levels for PFS. D: Cox regression parameter as a function of baseline ctDNA levels for OS.

Figure S5: Progression-free survival by VAF at baseline

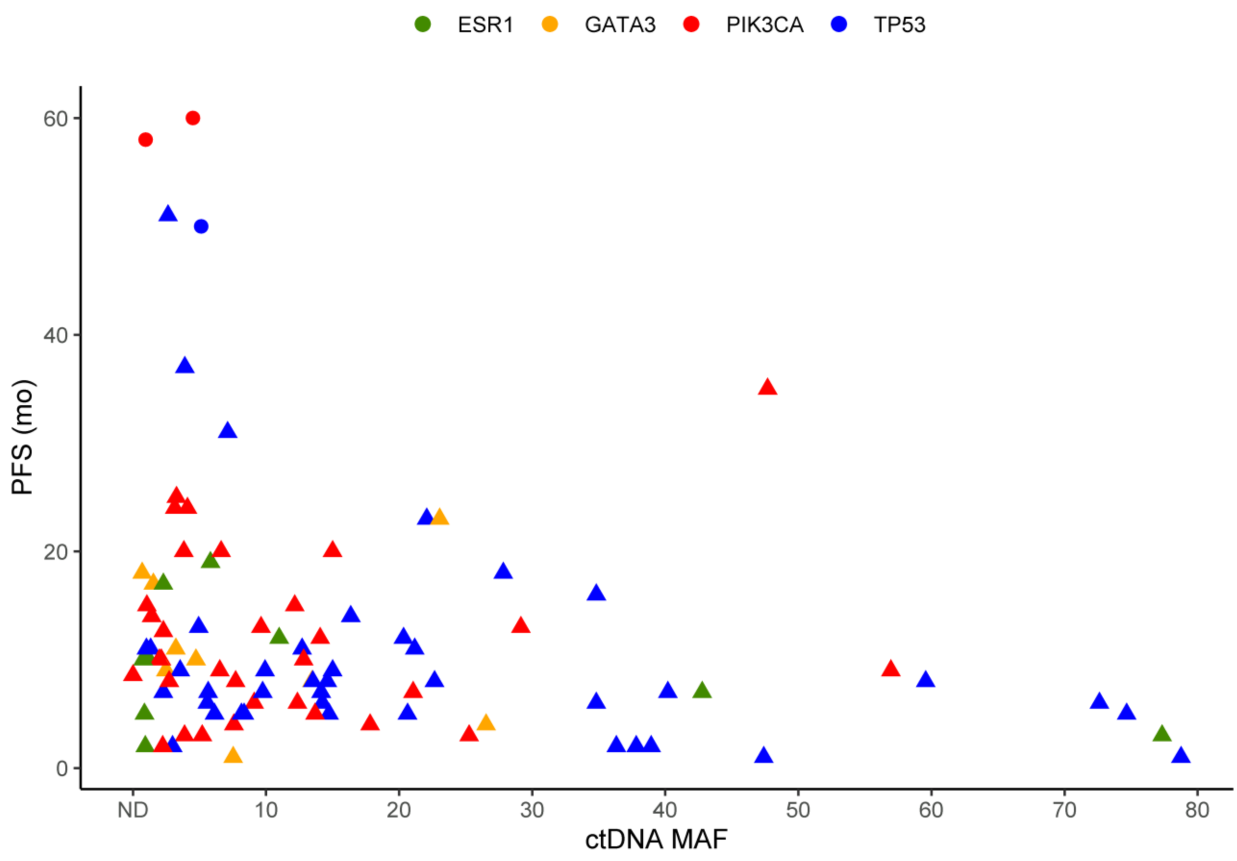

The four most frequently mutated genes are displayed. In patients with multiple mutated genes, the reporter variant is considered. N=3 patients were censored for PFS and displayed as circles. PFS events are displayed as triangles.

Figure S6: Overall survival according to *TP53* mutation status.

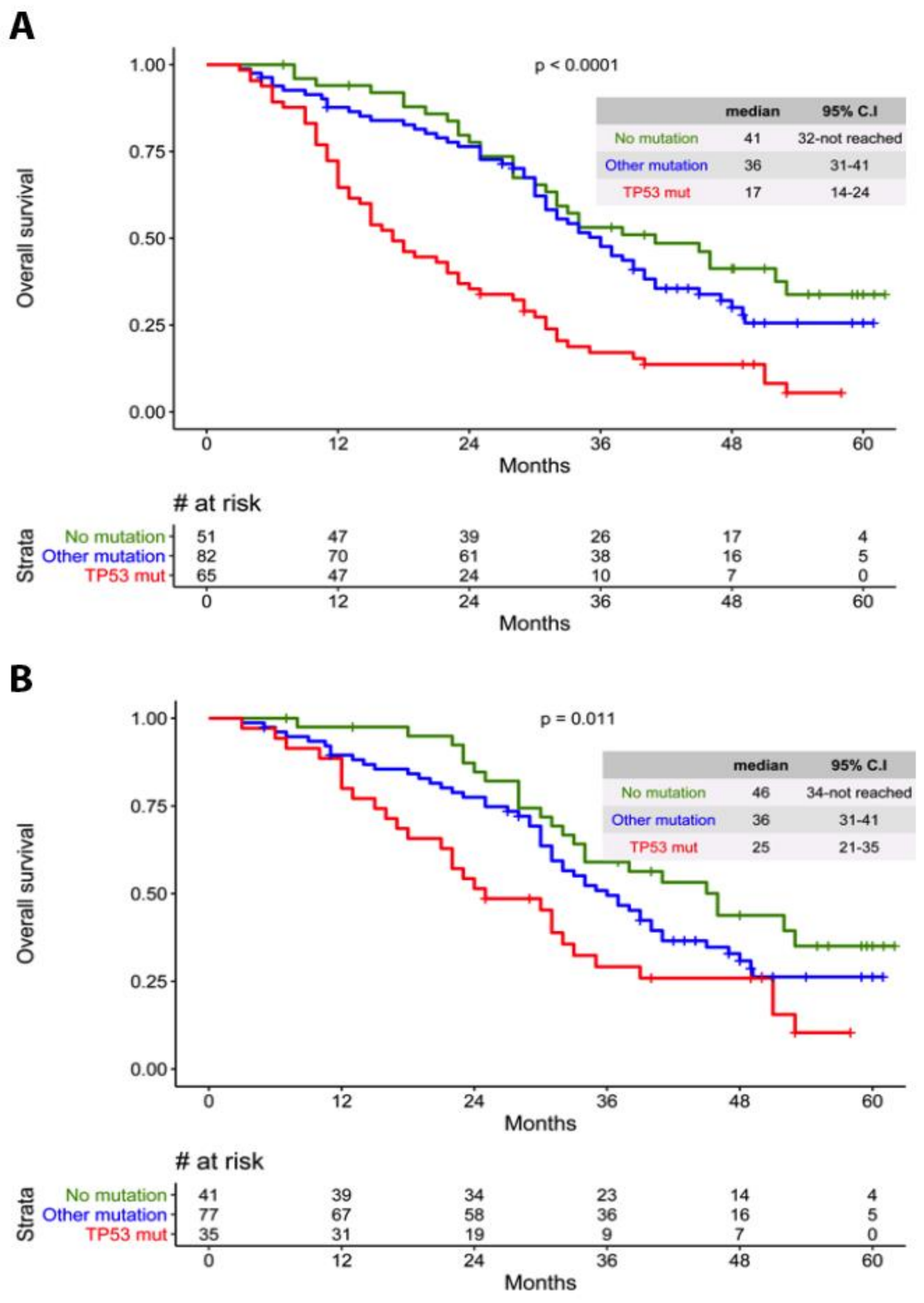

(A) Whole population (n=198). (B) HR+ HER2- subgroup (n=153)

Figure S7: Prognostic impact of CTC or ctDNA detection at 4 weeks

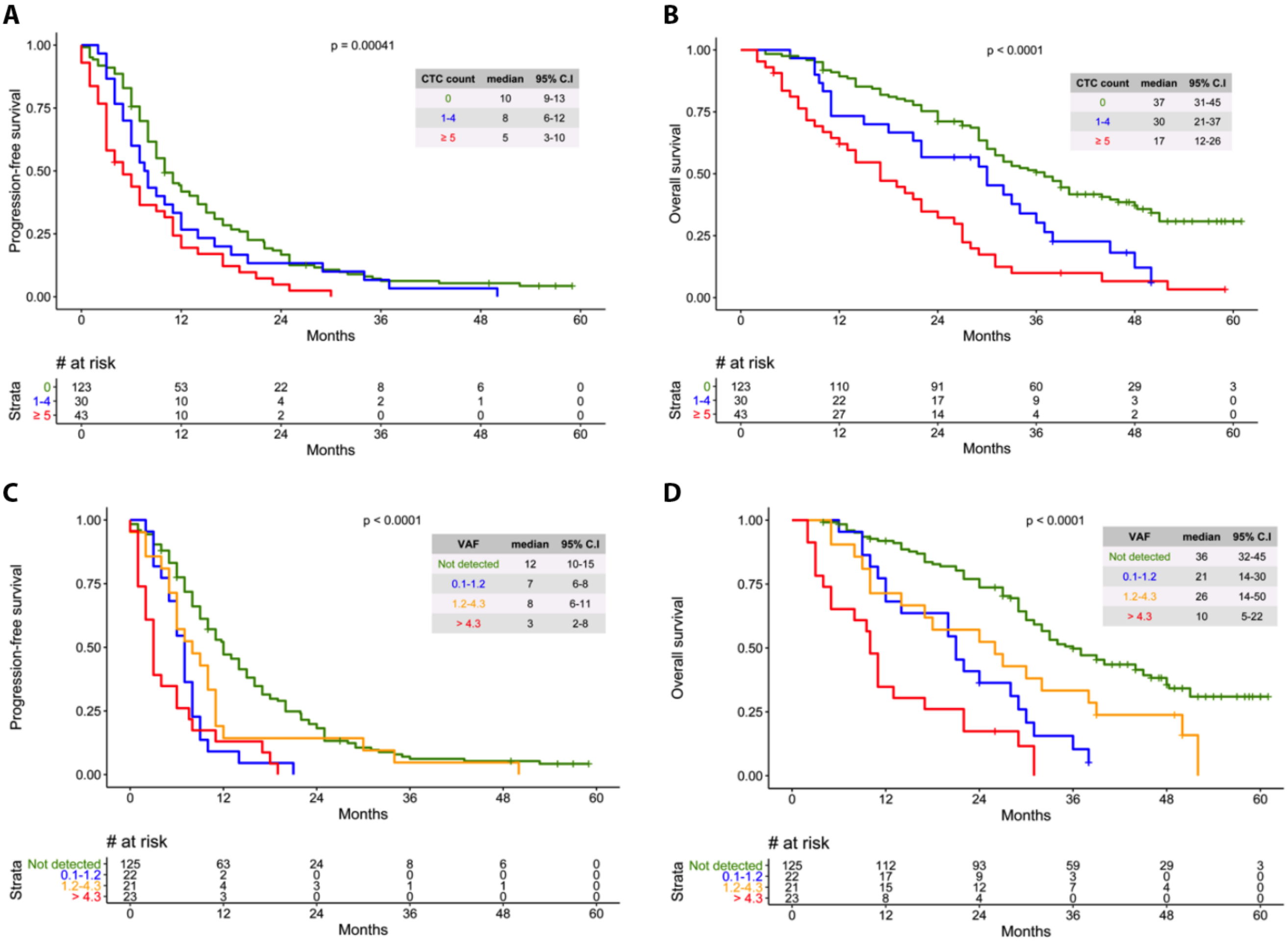

A) PFS by CTC count at 4 weeks. (B) OS by CTC count at 4 weeks. (C) PFS by ctDNA levels at 4 weeks. (D) OS by ctDNA levels at 4 weeks.

Figure S8: Variations of ctDNA values and CTC counts between baseline and 4 weeks

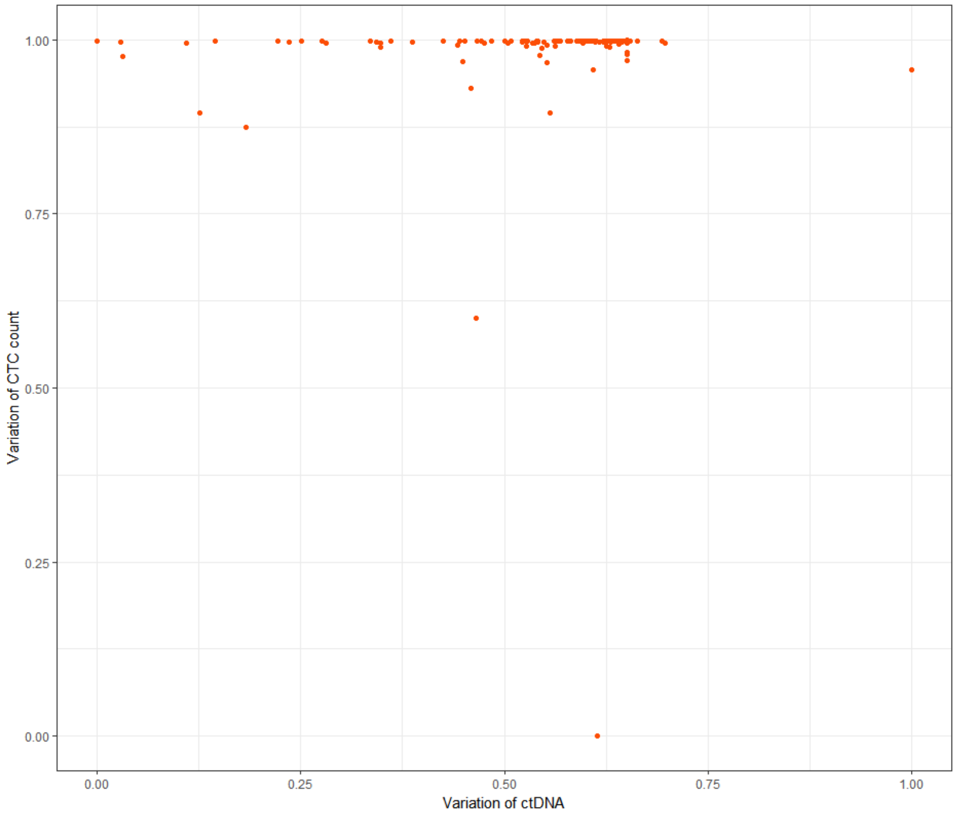

Normalization of the delta of ctDNA values and CTC counts between baseline and 4 weeks performed with the use of a Min-Max scaling.
