# Supplementary material for: Multimodal liquid biopsy for early monitoring and outcome prediction of chemotherapy in metastatic breast cancer
Source: NPJ Breast Cancer. 2021 Sep 9;7:115. doi: 10.1038/s41523-021-00319-4 (PMC8429692; doi:10.1038/s41523-021-00319-4)
Supplement: Supplementary file 2 — Reporting Summary [file 41523_2021_319_MOESM2_ESM.pdf]

nature research

Corresponding author(s): Jean-Yves Pierga NPJBCANCER-01197R

Last updated by author(s): 2021/05/28

Reporting Summary

Nature Research wishes to improve the reproducibility of the work that we publish. This form provides structure for consistency and transparency in reporting. For further information on Nature Research policies, see our Editorial Policies and the Editorial Policy Checklist.

Statistics

For all statistical analyses, confirm that the following items are present in the figure legend, table legend, main text, or Methods section.

n/a	Confirmed	
☐	☒	The exact sample size (n) for each experimental group/condition, given as a discrete number and unit of measurement
☐	☒	A statement on whether measurements were taken from distinct samples or whether the same sample was measured repeatedly
☐	☒	The statistical test(s) used AND whether they are one- or two-sided<br>*Only common tests should be described solely by name; describe more complex techniques in the Methods section.*
☐	☒	A description of all covariates tested
☐	☒	A description of any assumptions or corrections, such as tests of normality and adjustment for multiple comparisons
☐	☒	A full description of the statistical parameters including central tendency (e.g. means) or other basic estimates (e.g. regression coefficient) AND variation (e.g. standard deviation) or associated estimates of uncertainty (e.g. confidence intervals)
☒	☐	For null hypothesis testing, the test statistic (e.g. F, t, r) with confidence intervals, effect sizes, degrees of freedom and P value noted<br>*Give P values as exact values whenever suitable.*
☒	☐	For Bayesian analysis, information on the choice of priors and Markov chain Monte Carlo settings
☒	☐	For hierarchical and complex designs, identification of the appropriate level for tests and full reporting of outcomes
☒	☐	Estimates of effect sizes (e.g. Cohen's d, Pearson's r), indicating how they were calculated

Our web collection on statistics for biologists contains articles on many of the points above.

Software and code

Policy information about availability of computer code

Data collection	All statistical analyses were done with R software, version 3.4.2 (R Foundation for Statistical Computing).
Data analysis	All statistical analyses were done with R software, version 3.4.2 (R Foundation for Statistical Computing).<br>The source R codes and Rdata for the figures are available for nonprofit research use as Supplementary software.

For manuscripts utilizing custom algorithms or software that are central to the research but not yet described in published literature, software must be made available to editors and reviewers. We strongly encourage code deposition in a community repository (e.g. GitHub). See the Nature Research guidelines for submitting code & software for further information.

Data

Policy information about availability of data

All manuscripts must include a data availability statement. This statement should provide the following information, where applicable:

- Accession codes, unique identifiers, or web links for publicly available datasets
- A list of figures that have associated raw data
- A description of any restrictions on data availability

Data availability
PFS, OS data, CTC counts and ctDNA values for individual participant after de-identification are publicly available in Supplementary Table S5 and Rdata. The sequence metrics used for ctDNA analysis are available in Supplementary Table S2.

Field-specific reporting

Please select the one below that is the best fit for your research. If you are not sure, read the appropriate sections before making your selection.

☒ Life sciences ☐ Behavioural & social sciences ☐ Ecological, evolutionary & environmental sciences

For a reference copy of the document with all sections, see nature.com/documents/nr-reporting-summary-flat.pdf

Life sciences study design

All studies must disclose on these points even when the disclosure is negative.

Sample size	The primary endpoint of the study was the change of circulating endothelial cells and CTCs during therapy. The number of subjects to be included was calculated in order to detect a hazard ratio of 1.6 for death or progression in patients with CEC counts at 4 weeks < 20 CEC/4ml as compared to patients with CEC counts ≥ 20 CEC/4ml. We estimated that patients would be equally distributed between the two groups. Assuming a six-month PFS of 75%, an enrollment period of approximately 18 months, and a minimum follow-up of 2 years, 185 patients were required to provide the study with a power of 80% with a two-sided log-rank test at a significance level of 5%. Taking into account 10% of missing CEC data at 4 weeks, at least 206 patients had to be included.
Data exclusions	For sequencing data analysis, for FFPE samples, UMIs were not considered. Only variants with an allelic frequency higher than 0.1% at a locus covered by at least 8 reads were reported (mapping quality at 20 and base quality at 13). For FFPE samples, a minimum threshold of 5% VAF was used to filter out the variants.
Replication	Archived tumor material of patients included at our instituttion (available for 38 patients) was retrospectively sequenced to evaluate if variants identified in plasma samples were also observed in the tumor.
Randomization	No randomization, Phase IV study
Blinding	Blinding not relevant as not randomized trial

Reporting for specific materials, systems and methods

We require information from authors about some types of materials, experimental systems and methods used in many studies. Here, indicate whether each material, system or method listed is relevant to your study. If you are not sure if a list item applies to your research, read the appropriate section before selecting a response.

Materials & experimental systems

n/a	Involved in the study	
☒	☐	Antibodies
☒	☐	Eukaryotic cell lines
☒	☐	Palaeontology and archaeology
☒	☐	Animals and other organisms
☐	☒	Human research participants
☐	☒	Clinical data
☐	☐	Dual use research of concern

Methods

n/a	Involved in the study	
☒	☐	ChIP-seq
☒	☐	Flow cytometry
☒	☐	MRI-based neuroimaging

Human research participants

Policy information about studies involving human research participants

Population characteristics	Patients with triple negative or hormone receptors (HR) positive, HER2-negative metastatic breast cancer were enrolled in this study. Other main inclusion criteria were written informed consent, age > 18 years old, performance status (PS) of 0–2, life expectancy ≥ 3 months and no prior chemotherapy for mBC.<br>Exclusion criteria were prior chemotherapy for mBC, concomitant endocrine therapy or radiation therapy with curative intent for oligometastatic disease.
Recruitment	Patients used in this study were included in the multicentric prospective biomarker study “COMET” (NCT01745757) and were treated with weekly paclitaxel and bevacizumab as first line chemotherapy.
Ethics oversight	The study was approved by an ethics committee (Comité de Protection des Personnes “Ile de France VII”) in June 2012.

Note that full information on the approval of the study protocol must also be provided in the manuscript.

Clinical data

Policy information about clinical studies

All manuscripts should comply with the ICMJE guidelines for publication of clinical research and a completed CONSORT checklist must be included with all submissions.

Clinical trial registration	Registered under the following number NCT01745757.
Study protocol	https://clinicaltrials.gov/ct2/show/NCT01745757
Data collection	Out of the 510 patients enrolled in the whole COMET study between September 2012 and September 2014 from 14 french centers, analyses were performed on the 198 consecutive patients for whom CTC counts and ctDNA measurements were available at baseline.
Outcomes	PFS was defined as the time to disease progression or death of any cause. Overall survival was defined as the time to death, whatever the cause. Both PFS and OS were measured from the inclusion or the date of second sampling (4 weeks) for the analyses based on biomarker levels at 4 weeks (landmark analyses). PFS and OS curves based on separate or combined CTC and ctDNA values were estimated by the Kaplan–Meier method.

Dual use research of concern

Policy information about dual use research of concern

Hazards

Could the accidental, deliberate or reckless misuse of agents or technologies generated in the work, or the application of information presented in the manuscript, pose a threat to:

No	Yes	
☒	☐	Public health
☒	☐	National security
☒	☐	Crops and/or livestock
☒	☐	Ecosystems
☒	☐	Any other significant area

Experiments of concern

Does the work involve any of these experiments of concern:

No	Yes	
☒	☐	Demonstrate how to render a vaccine ineffective
☒	☐	Confer resistance to therapeutically useful antibiotics or antiviral agents
☒	☐	Enhance the virulence of a pathogen or render a nonpathogen virulent
☒	☐	Increase transmissibility of a pathogen
☒	☐	Alter the host range of a pathogen
☒	☐	Enable evasion of diagnostic/detection modalities
☒	☐	Enable the weaponization of a biological agent or toxin
☒	☐	Any other potentially harmful combination of experiments and agents
